# Tissue miRNA Combinations for the Differential Diagnosis of Adrenocortical Carcinoma and Adenoma Established by Artificial Intelligence

**DOI:** 10.3390/cancers14040895

**Published:** 2022-02-11

**Authors:** Péter István Turai, Zoltán Herold, Gábor Nyirő, Katalin Borka, Tamás Micsik, Judit Tőke, Nikolette Szücs, Miklós Tóth, Attila Patócs, Peter Igaz

**Affiliations:** 1 Department of Endocrinology, ENS@T Research Center of Excellence, Faculty of Medicine, Semmelweis University, H-1083 Budapest, Hungary; turai.peter_istvan@semmelweis-univ.hu (P.I.T.); nyiro.gabor1@med.semmelweis-univ.hu (G.N.); toke.judit@med.semmelweis-univ.hu (J.T.); szucs.nikolette@med.semmelweis-univ.hu (N.S.); toth.miklos@med.semmelweis-univ.hu (M.T.); 2 Department of Internal Medicine and Oncology, Faculty of Medicine, Semmelweis University, H-1083 Budapest, Hungary; 3 MTA-SE Molecular Medicine Research Group, Eötvös Loránd Research Network, H-1083 Budapest, Hungary; 4 Division of Oncology, Department of Internal Medicine and Oncology, Faculty of Medicine, Semmelweis University, H-1083 Budapest, Hungary; herold.zoltan@med.semmelweis-univ.hu; 5 Department of Laboratory Medicine, Faculty of Medicine, Semmelweis University, H-1089 Budapest, Hungary; patocs.attila@med.semmelweis-univ.hu; 6 2nd Department of Pathology, Semmelweis University, H-1091 Budapest, Hungary; borka.katalin@med.semmelweis-univ.hu; 7 1st Department of Pathology and Experimental Cancer Research, Semmelweis University, H-1088 Budapest, Hungary; micsik.tamas@med.semmelweis-univ.hu; 8 MTA-SE Hereditary Tumors Research Group, Eötvös Loránd Research Network, H-1122 Budapest, Hungary; 9 Department of Molecular Genetics, National Institute of Oncology, H-1122 Budapest, Hungary

**Keywords:** adrenocortical carcinoma, adenoma, adrenal, tissue, microRNA, biomarker, artificial intelligence, neural network

## Abstract

**Simple Summary:**

The histological differential diagnosis of adrenocortical adenoma and carcinoma is difficult and requires great expertise. MiRNAs were shown to be useful for the differential diagnosis of benign and malignant tumors of several organs, and several findings have suggested their utility in adrenocortical tumors as well. Here, we have selected tissue miRNAs based on the literature search, and used machine learning to identify novel clinically applicable miRNA combinations. Combinations with high sensitivity and specificity (both over 90%) have been identified that could be promising for clinical use. Besides being a useful adjunct to histological examination, these miRNA combinations could enable preoperative adrenal biopsy in patients with adrenal tumors suspicious for malignancy.

**Abstract:**

The histological analysis of adrenal tumors is difficult and requires great expertise. Tissue microRNA (miRNA) expression is distinct between benign and malignant tumors of several organs and can be useful for diagnostic purposes. MiRNAs are stable and their expression can be reliably reproduced from archived formalin-fixed, paraffin-embedded (FFPE) tissue blocks. Our purpose was to assess the potential applicability of combinations of literature-based miRNAs as markers of adrenocortical malignancy. Archived FFPE tissue samples from 10 adrenocortical carcinoma (ACC), 10 adrenocortical adenoma (ACA) and 10 normal adrenal cortex samples were analyzed in a discovery cohort, while 21 ACC and 22 ACA patients were studied in a blind manner in the validation cohort. The expression of miRNA was determined by RT-qPCR. Machine learning and neural network-based methods were used to find the best performing miRNA combination models. To evaluate diagnostic applicability, ROC-analysis was performed. We have identified three miRNA combinations (*hsa-miR-195* + *hsa-miR-210* + *hsa-miR-503*; *hsa-miR-210* + *hsa-miR-375* + *hsa-miR-503* and *hsa-miR-210* + *hsa-miR-483-5p* + *hsa-miR-503*) as unexpectedly good predictors to determine adrenocortical malignancy with sensitivity and specificity both of over 90%. These miRNA panels can supplement the histological examination of removed tumors and could even be performed from small volume adrenal biopsy samples preoperatively.

## 1. Introduction

Adrenal tumors are relatively frequent with a prevalence of 4.2% in high-resolution abdominal imaging studies [1]. Among adrenocortical tumors, adrenocortical carcinoma (ACC) has a poor prognosis, as less than a third of the patients survive at least 5 years [2,3,4]. Although ACC is the rarest among adrenal tumors, with an annual incidence of 0.7–2/million, it is included in the differential diagnosis of any incidentally discovered adrenal mass [3]. Adrenocortical adenoma (ACA) is the most frequent diagnosis (49–69% in surgical series) among adrenal tumors [5]. In addition to tumors of the adrenal cortex, myelolipoma, which is invariably benign and contains fat and bone marrow elements, and pheochromocytoma, of an adrenal medullary origin causing severe blood pressure fluctuations, may also occur [5]. Adrenal glands often harbor metastasis from distinct malignancies; moreover, adrenocortical hyperplasia, adrenal cyst, adrenal hemorrhage, and, very rarely, adrenal lymphoma and adrenal tuberculosis should also be kept in mind as potential adrenal pathologies [6]. The differentiation of adrenocortical adenoma and carcinoma is often challenging.

Medical imaging is especially helpful in establishing the diagnosis of adrenocortical malignancy. Tumor size, density, heterogeneity, irregular borders and necrosis are assessed on CT (computed tomography), and there are also options for further imaging, e.g., washout CT, MRI (magnetic resonance imaging) or ^18^FDG-PET-CT (^18^fluorodeoxyglucose-positron emission tomography-CT) [5]. Still there is no preoperative blood-borne molecular marker of malignancy. Urinary steroid metabolomics can be helpful [7], but it is not widely available.

The histological examination of adrenal tumors (including the Weiss-score and modified Weiss-score) is difficult and requires great expertise. Moreover, significant interobserver variability and a lack of accuracy in borderline cases are known limitations [8]. Mainly due to the difficulty of histological examinations, a biopsy of adrenal tumors is not recommended in routine practice and according to the current guidelines [3,5], as it would be difficult to determine malignancy from a small amount of tissue obtained, and there is a potential risk of complications (bleeding, pneumothorax) and maybe tumor dissemination as well [9,10]. The risk of complications linked to adrenal biopsy is not very high (2.5%), but it has only a sensitivity of 70% for diagnosing ACC [11,12].

For all these reasons there is a great need for additional markers that can help determine the biological behavior of adrenocortical tumors.

MicroRNAs (miRNA, miR) have long been one of the cornerstones of biomarker research [13]. MiRNAs are 19–25 nucleotide long evolutionary conserved single stranded non-coding RNA molecules, most often encoded by their own genes. MiRNAs are the epigenetic regulators of RNA interference as they regulate up to 30–60% of human genes at the post-transcriptional level—without altering the very sequence of DNA [14].

miRNAs exert their inhibitory functions on translation via binding to the 3′ untranslated region (UTR) of their target mRNA in the cytoplasm [15]. Besides, it was shown that miRNAs might act within the cell nucleus by the modification of histone proteins and transcription itself [16]. Biological functions of miRNAs have been characterized from abundant sources [17,18,19]. In tumors, both overexpressed (oncogenic) and underexpressed (tumor suppressor), miRNAs are known for acting in a tissue specific fashion [20,21,22]. From a biomarker research point of view the two most important features of miRNAs are their exceptional stability and reproducibility from fresh frozen tissue, FFPE (formalin-fixed, paraffin-embedded) samples or even from biofluids (e.g., from blood), and their marked tissue/cell and disease specificity [23,24]. Currently, there are about 2500 known human miRNAs and only a minor part of them has been described in the pathogenesis of adrenocortical tumors [25,26,27,28,29].

The long-lasting quest for a legit biomarker of adrenocortical carcinoma set our research group to design novel miRNA combination panels as markers of malignancy. Based on the current literature and bolstered by the state-of-art biostatistics tools, such as artificial intelligence (AI) implemented through machine learning and neural networks, our aim was to establish miRNA models with high sensitivity and specificity applicable for clinical use.

## 2. Materials and Methods

### 2.1. Tissue Collection and Ethics Approval

A total of 31 ACC, 32 ACA (Table 1) and 10 normal adrenal cortex (NAC) FFPE samples were recruited in the study. NAC samples were included only to investigate whether there are differences in the expression of the selected microRNAs between normal, benign, and malignant adrenocortical tissues. All samples were histologically confirmed by adrenal expert pathologists. Only specific parts of the blocks were dissected for RNA isolation. NAC samples were obtained from patients undergoing total nephrectomy for kidney tumors (females: 5, males: 5, mean age: 36.2 and 55.8, respectively). The discovery cohort was comprised of 10 ACA, 10 ACC, 10 NAC and the independent validation cohort contained another 21 ACC and 22 ACA FFPE samples (Table S1).

The study was approved by the Ethical Committee of the Hungarian Health Council. All experiments were performed in accordance with applicable guidelines and regulations and informed consent was obtained from the involved patients.

### 2.2. Literature Search

Literature search was performed in the PubMed database (https://pubmed.ncbi.nlm.nih.gov/) using the following search terms: adrenocortical carcinoma; adrenocortical cancer; adrenal cancer; adrenal tumor; and microRNA. Only original articles were selected. Most microRNAs included have been described as differentially expressed by multiple studies. We have selected 16 differentially expressed miRNAs to be included in our study (Table 2).

This list includes miRNAs that are extensively described in the literature to be important in adrenocortical tumor pathogenesis or differential diagnosis (such as *hsa-miR-195 or hsa-miR-483-5p, or hsa-miR-503*) [30,34,36,37,38,39,42,49,50,51], and also miRNAs where there is only limited evidence of pathogenic relevance. By including more miRNAs to be tested by artificial intelligence, we aimed to increase the chance of finding well-performing miRNA combinations.

### 2.3. Sample Processing and RNA Isolation

Total RNA was isolated by RecoverAll Total Nucleic Acid Isolation Kit for FFPE (catalog number: AM1975, Thermo Fisher Scientific, Waltham, MA, USA). As a spike-in control for isolation efficiency we used 1 μL of 0.002 fmol/µL *syn-cel-miR-39-3p* according to the manufacturer’s protocol for miRCURY LNA RNA Spike-in kit (Qiagen GmbH, Hilden, Germany, catalog number: 339390) and was added before the nucleic acid isolation step. Total RNA quantity was measured by NanoDrop 2000 Spectrophotometer (Thermo Fisher Scientific, Waltham, MA, USA) after isolation and Qubit 4 Fluorometer with Qubit™ hsRNA Assay Kit (Thermo Fisher Scientific, Waltham, MA, USA) before reverse transcription. Total RNA was stored at −80°C until further processing.

### 2.4. Analysis of the miRNA Panel Expression by Real-Time RT-qPCR

A 2-step process for RT-qPCR was used. Each sample was processed separately for all miRNA targets. Ten nanograms of isolated total RNA was used in individual RT reactions.

First, TaqMan miRNA Reverse Transcription Kit (catalog number: 4366596, Thermo Fisher Scientific, Waltham, MA, USA) and individual TaqMan MiRNA Assay primer mixes (catalog number: 4427975, Thermo Fisher Scientific, Waltham, MA, USA) were used to reverse-transcribe total RNA. The expression of *hsa-miR-7 (*ID: 000386*), hsa-miR-9 (*ID: 000583*), hsa-miR-21 (*ID: 000397*), hsa-miR-195 (*ID: 000494*), hsa-miR-205 (*ID: 000509*), hsa-miR-210 (*ID: 000512*), hsa-miR-214 (*ID: 002306*), hsa-miR-335 (*ID: 000546*), hsa-miR-375 (*ID: 000564*), hsa-miR-431 (*ID: 001979*), hsa-miR-483-3p (*ID: 002339*), hsa-miR-483-5p* (ID: 002338), *hsa-miR-497* (ID: 001043)*, hsa-miR-503 (*ID: 001048*), hsa-miR-508 (*ID: 001052*),* and *hsa-miR-511 (*ID: 001111*)* were measured, and as an internal control *RNU48* (ID: 001006) along with *cel-miR-39* (ID: 000200) as an external control were used.

For quantification, TaqMan Fast Advanced Master Mix (catalog number: 4444963, Thermo Fisher Scientific, Waltham, MA, USA), with the matching probe mixes on a Quantstudio 7 Flex Real-Time PCR System (Thermo Fisher Scientific, Waltham, MA, USA) according to the manufacturer’s protocol, was used. Negative control reactions contained no cDNA templates, and all samples were measured in triplicate. We used 0,67 µL of undiluted cDNA as template.

After analysis of the miRNA panel expression by real-time RT-qPCR on the discovery cohort, we proceeded to validate our best performing combinations by carrying out another set of real-time RT-qPCR measurements on an independent validation cohort, but with a further refined group of miRNAs: *hsa-miR-9 (*ID: 000583*), hsa-miR-195 (*ID: 000494*), hsa-miR-210 (*ID: 000512*), hsa-miR-375 (*ID: 000564*), hsa-miR-483-3p (*ID: 002339*), hsa-miR-483-5p* (ID: 002338), *hsa-miR-497* (ID: 001043)*, hsa-miR-503 (*ID: 001048*)*, and *hsa-miR-508 (*ID: 001052*)*.

### 2.5. Statistical Analysis

Statistical analysis was carried out with R for Windows version 4.1.1 (R Foundation for Statistical Computing, 2021, Vienna, Austria). Normalization of miRNAs was performed with the ΔCt method, in which geometric means of intrinsic “housekeeping gene” (*RNU48*) and extrinsic spike-in (*cel-miR-39*) served as controls (R package NormqPCR). Down-regulated miRNAs, when presented with no measurable Ct values, were omitted. The order of miRNAs that played prominent role in the group classification of the samples was determined by the random forest method, using the importance measure ‘mean decrease in accuracy’ (R package randomForest), which was used to strengthen relationships already known from the literature [54]. The possibility of automatic classification of samples into ACA or ACC groups was tested by machine learning methods (R packages nnet and caret) [55,56]. The classification efficiency of possible miRNA combinations was examined by neural network-based, 90–10% random learner-tester cross validation consisting of 10-10-10 known ACC, ACA, and NAC samples. A hidden-layer neural network-based statistical model was created that randomly selected 9-9-9 samples per group from 10-10-10 histological specimens (learner data set). Classification efficacy of the model was tested on the remaining 1-1-1 samples (tester data set). By repeating this step 1000 times, we were able to determine the miRNA combinations, which had high specificity and sensitivity for group classification. The analysis was also performed both on all 30 samples from all three groups and on the 20 samples from benign and malignant adrenal tumors alone as well. Twenty-four models with at least 90% classification capability were selected for validation of subsequent machine learning-based classification (Table 3).

During validation, the same ACA and ACC samples were used as previously, and the 43 unknown samples were classified individually, with 10,000 iterations each. The final estimated group classification of the sample was determined by selecting the most common value (>50%) from the 10,000 estimates.

Sensitivity and specificity for each model were determined—after revealing the benign or malignant histological diagnosis of each sample—by comparing the estimated and the actual groupings from the models. At this point, as a technical step, the ACA group was designated as the “control” group and the ACC group as the “patient” group. Based on the differences between the two classifications, we determined the number of correct results (true positives and negatives), false positive (benign tumor instead of malignant tumor) and false negative (malignant tumor instead of benign tumor) results.

The percentage of correct classifications in the ACA group and the correct classification were compared and plotted by ROC analysis (R package pROC) [57]. Additional epidemiological measures (e.g., area under curve) were determined using the true group classifications and the percentages of the estimated classification in the ROC analysis.

## 3. Results

### 3.1. miRNA Expression in the Discovery Cohort by RT-qPCR

**Figure 1:**
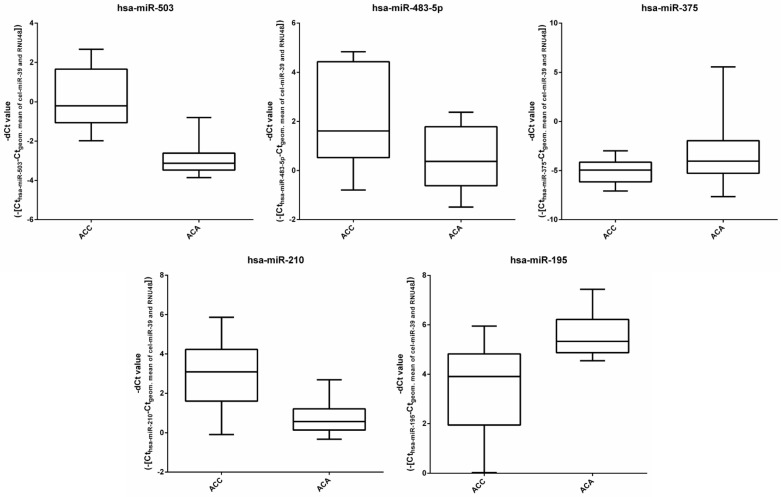
Box plots representing the expression of the top five miRNAs relative to the geometric means of cel-miR-39 and RNU48 in ACA and ACC samples. The top 5 selected miRNAs contributing to the best performing three models were determined based on artificial intelligence.

RT-qPCR was performed on 10-10-10 known ACA, ACC, NAC FFPE tissue samples in the discovery cohort. The list of selected miRNAs is presented in Table 2. Random forest results revealed that hsa-miR-503, hsa-miR-483_3p, hsa-miR-195, hsa-miR-375 and hsa-miR-483_5p were the top 5 miRNAs to properly group the 30 samples into their respective groups. (Figure 1 presents box plots representing the expression of these five miRNA in ACA and ACC.) The best performing miRNA combinations (statistical models) were selected by neural network-based, 90–10% random learner-tester cross validation. Twenty-four statistical models (Table 3) with at least 90% grouping capability were selected for validation. These 24 models contain the following miRNAs: hsa*-miR-9, hsa-miR-195, hsa-miR-210, hsa-miR-375, hsa-miR-483-3p, hsa-miR-483-5p, hsa-miR-497, hsa-miR-503,* and *hsa-miR-508*.

### 3.2. Diagnostic Performance of the miRNA Models by RT-qPCR

**Figure 2:**
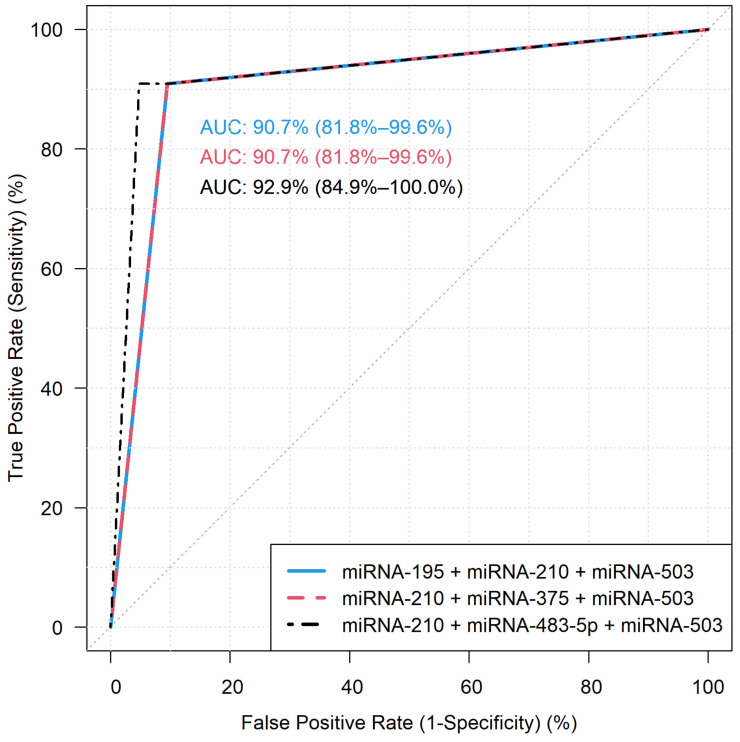
ROC curves of the best performing three miRNA combinations. Model 9: *hsa-miR-195 + hsa-miR-210 + hsa-miR-503* (left upper corner), model 16: *hsa-miR-210 + hsa-miR-375 + hsa-miR-503* (right upper corner), model 17: *hsa-miR-210 + hsa-miR-483-5p + hsa-miR-503* (down). AUC: area under curve.

In total, 43 independent FFPE samples (22 ACA and 21 ACC) were measured in the validation cohort by RT-qPCR to establish the utility of selected miRNA combinations as markers of malignancy. Table 4 presents the sensitivity, specificity, area under curve, positive and negative predictive values of the 24 models. Among these, 3 models yielded sensitivity and specificity both over 90%: model 9 (*hsa-miR-195 + hsa-miR-210 + hsa-miR-503*), model 16 (*hsa-miR-210 + hsa-miR-375 + hsa-miR-503*) and model 17 (*hsa-miR-210 + hsa-miR-483-5p + hsa-miR-503*) (Figure 2). False negative (V14, V19) and false positive (V33) samples are marked in Table S1. These samples were commonly missed by the three best performing models, whereas Sample V38 has been recognized by Model 17, and not by the two other models. We could not find common or peculiar features in the falsely classified samples. The values for individual miRNAs are presented in Table 5. These combination-based predictions are clearly superior to the diagnostic performance of individual miRNAs.

## 4. Discussion

The histological diagnosis of adrenocortical tumors is challenging. In this study, we assessed the applicability for various miRNA combinations established by an artificial intelligence approach (machine learning and neural networks) that could reliably be utilized as markers of adrenocortical malignancy.

Sixteen miRNAs were included in our study, based on the literature search, but the established miRNA combinations include only 5 of these (*hsa-miR-195, hsa-miR-210, hsa-miR-375, hsa-miR-483-5p,* and *hsa-miR-503*). Not surprisingly, this 5-miRNA set includes the miRNAs that have been described in most adrenocortical tumor studies as differentially expressed between benign and malignant tumors.

*Hsa-miR-195* was shown to be underexpressed in ACC compared to ACA in various studies [30,34,36,37,38,39]. Furthermore, the underexpression of *hsa-miR-195* was associated with poor outcome, and lower circulating levels of *hsa-miR-195* tended to be correlated with a larger tumor size [30,36,38]. On the other hand, the up-regulation of *hsa-miR-195* decreased cell proliferation in human NCI-H295R ACC cells [34]. The gene for *hsa-miR-195* is located within the genomic region of 17p13, that was shown to be frequently lost in adrenocortical tumors [58].

*Hsa-miR-210* is a general hypoxamiR as it was shown to be involved in tumor hypoxia, thereby, the overexpression of *hsa-miR-210* seems to be a common event in various tumors [59]. *Hsa-miR-210* is regulated by the hypoxia-inducible factor 1α (HIF1α), an important factor in antitumoral therapy resistance [60,61,62]. It was shown to be overexpressed in ACC compared to ACA and NAC in multiple studies [34,39,42,43], and also significantly overexpressed in ACC with distant metastases [38]. High expression of *hsa-miR-210* was associated with poor prognosis [47].

*Hsa-miR-375* was shown to be significantly underexpressed in ACC and ACA compared to NAC in our previous study [42]. It targets certain oncogenes, such as *AEG-1/MTDH, PDK1, YWHAZ/14-3-3ζ, YAP* and *JAK2*, in multiple types of carcinomas [63,64,65,66,67]. Reciprocal action between Wnt–β-catenin signaling and *hsa*-*miR-375* has been proposed [68]. The Wnt–β-catenin pathway is an important factor in the pathogenesis of ACC [69,70]. It was surprising that this miRNA has been included in the models by artificial intelligence, and even in one of the best performing combinations (Model 16).

Overexpressed *hsa-miR-483-5p* is considered to be the best marker of adrenocortical malignancy [26,31,33,35,36,41,47,48,49,69,70]. However, we have recently shown its limitation in the differentiation of ACC and adrenal myelolipoma [71]. *Hsa-miR-483-5p* is coexpressed with the insulin-like growth factor 2 (*IGF2*) from the same locus at 11p15.5 [37]. Overexpression of *IGF2* mRNA is a main feature of ACC [72,73]. N-myc downstream-regulated gene family members 2 and 4 (*NDRG2* and *NDRG4*) were identified as targets of *miR-483-5p*, and their expression was inversely correlated with *miR-483-5p* [74]. *Hsa-miR-483-5p* is also an interesting example of miRNA’s tissue and disease specificity as it has been shown to be down-regulated in Wilms tumors and glioma cells, suggesting its tumor suppressor activity in these tumors and tissues [75,76].

*Hsa-miR-503* has also been described in several adrenal tumor studies [34,36,38,42,43]. Its pathogenic role was also proposed in other malignancies [77,78]. A larger tumor size has been shown to correlate with the overexpression of *hsa-miR-503*, and also, a significant correlation with Weiss-criteria, clinical outcome and survival was revealed [34,38]*. Hsa-miR-503* has previously been described as a direct cell cycle and differentiation regulator in different cell lines [79,80].

The three best-performing miRNA combinations yielded clearly superior sensitivity and specificity values than the individual miRNAs included in the combinations (Table 4 vs. Table 5.) and also than the previous literature data for individual miRNAs (e.g., sensitivity–specificity: 68.7–93.7; 73.7–100 for *hsa-miR-195* and for *hsa-miR-483-5p*, respectively [33]). Some literature data, however, show comparable, or even better diagnostic performance data than our combinations. For example, in our previous study, the combination of *hsa-miR-511* and *hsa-miR-503* was associated with 100% sensitivity and 97% specificity [42], and in Feinmesser’s study a 100% sensitivity and 96% specificity of the *hsa-miR-497* and *hsa-miR-34a* combination was noted [38]. In most previous studies, however, smaller cohorts were included, (e.g., only 7 and 17 ACC samples included in the two above mentioned studies, respectively [38,42]). Different cohort compositions, platforms and statistical methods might also be accounted for these differences.

Our study certainly has limitations. These include the limited set of miRNAs examined and the sizes of the cohorts that are larger than in most previous studies but should still be augmented to assess the clinical utility of the markers identified. Moreover, we performed our measurements on FFPE samples in a retrospective setup, hence the clinical utility of these miRNA combinations should further be examined on fresh frozen samples in a prospective manner.

Using small sample sizes in machine learning techniques can lead to biased machine learning performance estimates. To overcome this type of bias, it is recommended to use a different, new dataset for validation. In our study, both the baseline and validation cohorts consist of different patients; therefore, our results do not suffer from this type of bias.

Another type of bias can be introduced from using specific types of cross validation. It was previously reported that using nested types of cross-validation produce unbiased and robust results [81]. The 90%–10% random learner-tester cross-validation used in our study belongs to the nested cross-validation family.

The sensitivity and specificity values of the three best performing biomarker combinations appear to be promising for clinical introduction. Besides a useful adjunct to histological analysis of surgically resected tumor specimens, the possible testing of these microRNA panels on adrenal biopsy samples might also be envisaged. Adrenal biopsy is currently not recommended in the work-up of adrenal tumors, only in exceptional cases, mainly due to the difficulties of histological analysis, but there are also some possible complications [3,5,11,12]. If the diagnosis of malignancy could be reliably established by using these microRNA panels from small biopsy samples, this might even broaden the use of adrenal biopsy in preoperative diagnosis and the current recommendations might be revisited.

## 5. Conclusions

In this study, novel miRNA marker combinations have been established by artificial intelligence-based methods showing high sensitivity and specificity that could aid in the differential diagnosis of benign and malignant adrenocortical tissue specimens. The clinical utility of these biomarkers should be further validated in even larger sample cohorts, and their potential use on biopsy samples might also be evaluated. Prospective analysis on fresh frozen samples is also warranted. These miRNA combinations could help postoperative histological diagnosis.

## 6. Patents

Claims for patenting the three best performing biomarker combinations have been submitted to the Hungarian Intellectual Property Office (P2200007).

## Figures and Tables

**Table 1:** Clinical and main pathological characteristics of the tumor samples included. F: female, M: male, NF: non-functioning, DHEAS: dehydroepiandrosterone sulfate, DOC: 11-Deoxycorticosterone, ND: no data.

Cohort/Samples	Sex	Mean Age at Sample Taking (Years)	Mean Tumor Size (mm)	Ki-67 (%)	ENSAT Stage	Hormonal Activity
Discovery ACA	10 F	47.5	33.9	-	-	7 cortisol 3 NF
Discovery ACC	6 F 4 M	45.2	96.2	10–15 (1–40)	5 II 5 III	3 cortisol 5 NF 1 DOC 1 DOC + cortisol + estradiol
Validation ACA	17 F 5 M	53.9	35	-	-	11 cortisol 10 NF 1 DHEAS
Validation ACC	14 F 7 M	55.4	102	25–30 (8–50)	1 I 4 II 5 III 11 IV	7 cortisol 11 NF 2 cortisol + DHEAS 1 cortisol + androgen

**Table 2:** List of selected, differentially expressed miRNAs based on literature search that were included in our study.

miRNAs	Expression in ACC	References
*hsa-miR-7*	Down-regulated	[30,31]
*hsa-miR-9*	Up-regulated	[32,33]
*hsa-miR-21*	Up-regulated	[34,35]
*hsa-miR-195*	Down-regulated	[30,34,36,37,38,39]
*hsa-miR-205*	Down-regulated	[40,41]
*hsa-miR-210*	Up-regulated	[34,39,42,43]
*hsa-miR-214*	Down-regulated	[38,42,44]
*hsa-miR-335*	Down-regulated	[36,38,45]
*hsa-miR-375*	Down-regulated	[42]
*hsa-miR-431*	Down-regulated	[44,46]
*hsa-miR-483-3p*	Up-regulated	[34,38,47,48]
*hsa-miR-483-5p*	Up-regulated	[30,34,36,38,39,49,50,51]
*hsa-miR-497*	Down-regulated	[34,38]
*hsa-miR-503*	Up-regulated	[38,42]
*hsa-miR-508*	Up-regulated	[36,44,52]
*hsa-miR-511*	Down-regulated	[42,44,53]

**Table 3:** The 24 miRNA combination models used in the validation cohort.

Model Number	miRNA Combination
1	*hsa-miR-9 + hsa-miR-375*
2	*hsa-miR-9 + hsa-miR-503*
3	*hsa-miR-375 + hsa-miR-503*
4	*hsa-miR-210 + hsa-miR-503*
5	*hsa-miR-375 + hsa-miR-497*
6	*hsa-miR-483-3p + hsa-miR-503*
7	*hsa-miR-503 + hsa-miR-508*
8	*hsa-miR-195 + hsa-miR-503 + hsa-miR-508*
9	*hsa-miR-195 + hsa-miR-210 + hsa-miR-503*
10	*hsa-miR-9 + hsa-miR-195 + hsa-miR-503*
11	*hsa-miR-9 + hsa-miR-210 + hsa-miR-503*
12	*hsa-miR-9 + hsa-miR-375 + hsa-miR-503*
13	*hsa-miR-9 + hsa-miR-483-3p + hsa-miR-503*
14	*hsa-miR-9 + hsa-miR-497 + hsa-miR-503*
15	*hsa-miR-195 + hsa-miR-375 + hsa-miR-497*
16	*hsa-miR-210 + hsa-miR-375 + hsa-miR-503*
17	*hsa-miR-210 + hsa-miR-483-5p + hsa-miR-503*
18	*hsa-miR-375 + hsa-miR-503 + hsa-miR-508*
19	*hsa-miR-375 + hsa-miR-483-3p + hsa-miR-503*
20	*hsa-miR-9 + hsa-miR-195 + hsa-miR-375 + hsa-miR-503*
21	*hsa-miR-9 + hsa-miR-210 + hsa-miR-483-5p + hsa-miR-503*
22	*hsa-miR-210 + hsa-miR-375 + hsa-miR-503 + hsa-miR-508*
23	*hsa-miR-375 + hsa-miR-483-5p + hsa-miR-503 + hsa-miR-508*
24	*hsa-miR-375 + hsa-miR-497 + hsa-miR-503 + hsa-miR-508*

**Table 4:** Diagnostic performance of the 24 miRNA combination models. The best performing three models are highlighted in bold.

Model Number	Sensitivity	Specificity	Area under Curve (AUC)	Negative Predictive Value	Positive Predictive Value
1	72.73%	42.86%	56.49%	57.14%	60.00%
2	72.73%	85.71%	81.17%	84.21%	75.00%
3	90.91%	85.71%	90.04%	86.96%	90.00%
4	86.36%	90.48%	88.42%	90.48%	86.36%
5	86.36%	66.67%	76.52%	73.08%	82.35%
6	72.73%	95.24%	86.15%	94.12%	76.92%
7	81.82%	90.48%	85.93%	90.00%	82.61%
8	86.36%	85.71%	87.34%	86.36%	85.71%
9	**90.91%**	**90.48%**	**90.69%**	**90.91%**	**90.48%**
10	68.18%	85.71%	78.90%	83.33%	72.00%
11	86.36%	85.71%	88.10%	86.36%	85.71%
12	86.36%	80.95%	83.66%	82.61%	85.00%
13	68.18%	90.48%	82.47%	88.24%	73.08%
14	77.27%	85.71%	80.84%	85.00%	78.26%
15	86.36%	66.67%	76.52%	73.08%	82.35%
16	**90.91%**	**90.48%**	**90.69%**	**90.91%**	**90.48%**
17	**90.91%**	**95.24%**	**92.86%**	**95.24%**	**90.91%**
18	90.91%	85.71%	90.04%	86.96%	90.00%
19	77.27%	90.48%	85.61%	89.47%	79.17%
20	86.36%	80.95%	85.50%	82.61%	85.00%
21	86.36%	80.95%	85.71%	82.61%	85.00%
22	90.91%	85.71%	90.04%	86.96%	90.00%
23	90.91%	85.71%	88.31%	86.96%	90.00%
24	90.91%	85.71%	89.39%	86.96%	90.00%

**Table 5:** Individual diagnostic performance of the miRNAs included in the 24 miRNA combination models.

miRNA	Sensitivity	Specificity	Area under Curve (AUC)	Negative Predictive Value	Positive Predictive Value
*hsa-miR-9*	54.55%	61.90%	59.52%	60.00%	56.52%
*hsa-miR-195*	86.36%	71.43%	78.90%	76.00%	83.33%
*hsa-miR-210*	68.18%	80.95%	76.41%	78.95%	70.83%
*hsa-miR-375*	81.82%	23.81%	53.68%	52.94%	55.56%
*hsa-miR-483-3p*	54.55%	90.48%	74.57%	85.71%	65.52%
*hsa-miR-483-5p*	81.82%	90.48%	86.15%	90.00%	82.61%
*hsa-miR-497*	86.36%	80.95%	83.66%	82.61%	85.00%
*hsa-miR-503*	81.82%	90.48%	86.15%	90.00%	82.61%
*hsa-miR-508*	59.09%	52.38%	58.33%	56.52%	55.00%

## Data Availability

Data are contained within the article or supplementary material. The data presented in this study are available in Table S1.

## Supplementary Materials

The following supporting information can be downloaded at: https://www.mdpi.com/article/10.3390/cancers14040895/s1, Table S1: Clinical and main pathological characteristics of the tumor samples
