# Supplementary material for: Tissue miRNA Combinations for the Differential Diagnosis of Adrenocortical Carcinoma and Adenoma Established by Artificial Intelligence
Source: Cancers (Basel). 2022 Feb 11;14(4):895. doi: 10.3390/cancers14040895 (PMC8870702; doi:10.3390/cancers14040895)
Supplement: Supplementary file 1 [file cancers-14-00895-s001.zip › cancers-1573047-supplementary.pdf]

Supplementary Table S1. Clinical and main pathological characteristics of the tumor samples included. F: female, M: male, NF: non-functioning, DHEAS: dehydroepiandrosterone sulfate, DOC: 11-Deoxycorticosterone, ND: no data. Bold: false positive (red) or false negative (blue) samples by the three best performing models.

Discovery cohort	Tumor type	Sex	Age at sampling	Tumor size (mm)	Ki-67 (%)	ENSAT stage	Hormonal activity
D1	ACA	female	32	36	-	-	Cortisol
D2	ACA	female	40	43	-	-	Cortisol
D3	ACA	female	62	50	-	-	Cortisol
D4	ACA	female	74	20	-	-	NF
D5	ACA	female	51	25	-	-	Cortisol
D6	ACA	female	71	15	-	-	NF
D7	ACA	female	30	25	-	-	Cortisol
D8	ACA	female	43	45	-	-	Cortisol
D9	ACA	female	38	35	-	-	Cortisol
D10	ACA	female	34	45	-	-	NF
D11	ACC	female	39	65	ND	II	Cortisol
D12	ACC	male	40	100	1-15	II	NF
D13	ACC	female	19	150	10	III	DOC
D14	ACC	male	48	200	2-5	III	NF
D15	ACC	female	37	60	2-5	III	DOC, cortisol, estradiol
D16	ACC	male	58	75	30	III	Cortisol
D17	ACC	female	73	65	30-40	II	NF
D18	ACC	male	39	85	5-10	II	NF
D19	ACC	female	53	62	5	II	NF
D20	ACC	female	46	100	ND	III	Cortisol
**Validation cohort**	**Tumor type**	**Sex**	**Age at sample taking**	**Tumor size (mm)**	**Ki-67 (%)**	**ENSAT stage**	**Hormonal activity**
V1	ACA	female	63	25	-	-	NF
V2	ACA	female	46	25	-	-	Cortisol
V3	ACA	female	45	30	-	-	Cortisol
V4	ACA	male	68	50	-	-	NF
V5	ACA	female	64	38	-	-	Cortisol
V6	ACA	female	39	25	-	-	Cortisol
V7	ACA	female	32	35	-	-	Cortisol
V8	ACA	male	72	40	-	-	NF
V9	ACA	female	31	25	-	-	Cortisol
V10	ACA	female	72	43	-	-	NF
V11	ACA	female	78	45	-	-	DHEAS
V12	ACA	female	45	30	-	-	Cortisol
V13	ACA	female	39	35	-	-	NF
**V14**	**ACA**	**female**	**50**	**52**	**-**	**-**	**Cortisol**
V15	ACA	female	60	32	-	-	NF
V16	ACA	female	49	25	-	-	Cortisol
V17	ACA	female	34	25	-	-	NF
V18	ACA	male	46	57	-	-	NF
**V19**	**ACA**	**male**	**50**	**30**	**-**	**-**	**NF**
V20	ACA	female	62	57	-	-	Cortisol
V21	ACA	female	69	30	-	-	NF

V22	ACA	male	71	18	-	-	Cortisol
V23	ACC	female	47	140	20-25	II	NF
V24	ACC	female	53	80	40-50	IV	Cortisol, androgen
V25	ACC	female	70	120	20	III	NF
V26	ACC	female	75	75	40-50	IV	Cortisol
V27	ACC	female	71	100	25	IV	NF
V28	ACC	female	46	200	43	IV	NF
V29	ACC	male	33	190	ND	II	Cortisol, DHEAS
V30	ACC	male	43	120	ND	IV	NF
V31	ACC	male	56	65	20-40	II	NF
V32	ACC	female	72	95	20-30	III	Cortisol
**V33**	**ACC**	**female**	**62**	**160**	**8**	**III**	**Cortisol**
V34	ACC	male	17	110	20-25	II	NF
V35	ACC	female	49	40	ND	I	NF
V36	ACC	female	61	80	20-30	III	Cortisol
V37	ACC	female	61	100	ND	IV	NF
V38	ACC	male	79	120	10	III	Cortisol, DHEAS
V39	ACC	female	55	75	20-25	IV	Cortisol
V40	ACC	male	48	61	10	IV	Cortisol
V41	ACC	female	62	60	40-50	IV	Cortisol
V42	ACC	female	55	55	25	IV	NF
V43	ACC	male	48	ND	ND	IV	NF
